# Prognostic and Treatment-Specific Predictive Implications of HER2 Expression in RAS Wild-Type Metastatic Colorectal Cancer: A Multicenter Retrospective Real-World Study

**DOI:** 10.3390/jcm15103979

**Published:** 2026-05-21

**Authors:** Özlem Özdemir, Damla Günenç, Halil Taşkaynatan, Pınar Peker, Emir Gökhan Kahraman, Sedat Biter, Semra Paydaş, Tuğba Önder, Öztürk Ateş, Muhammed Muhiddin Er, Murat Araz, Ahmet Melih Arslan, Hüseyin Salih Semiz, Nilüfer Avcı, İzzet Doğan, Akif Doğan, Teoman Şakalar, Timur Köse, Asuman Argon, Enver İlhan, Başak Doğanavşargil Yakut, Murat Sezak, Bülent Karabulut

**Affiliations:** 1 Department of Medical Oncology, Izmir Faculty of Medicine, University of Health Sciences, Izmir City Hospital, 35540 İzmir, Turkey; haliltaskaynatan@gmail.com (H.T.); emirgokhan@gmail.com (E.G.K.); 2 Department of Medical Oncology, Ege University Faculty of Medicine, 35100 İzmir, Turkey; pnnarrrr@gmail.com; 3 Department of Medical Oncology, Çukurova University Faculty of Medicine, 01330 Adana, Turkey; sedatb23@hotmail.com (S.B.); sepay@cu.edu.tr (S.P.); 4 Department of Medical Oncology, Dr. Abdurrahman Yurtaslan Ankara Oncology Training and Research Hospital, 06200 Ankara, Turkey; 5 Department of Medical Oncology, Necmettin Erbakan University Meram Faculty of Medicine, 42080 Konya, Turkey; 6 Department of Medical Oncology, Dokuz Eylül University Faculty of Medicine, 35340 İzmir, Turkey; 7 Department of Medical Oncology, Medicana Hospital, 16140 Bursa, Turkey; 8 Department of Medical Oncology, Acıbadem Bakırköy Hospital, 34140 İstanbul, Turkey; 9 Department of Medical Oncology, Şehit Prof. Dr. İlhan Varank Training and Research Hospital, 34785 İstanbul, Turkey; 10 Department of Medical Oncology, Necip Fazıl City Hospital, 46050 Kahramanmaraş, Turkey; 11 Department of Biostatistics and Medical Informatics, Ege University, 35100 İzmir, Turkey; 12 Department of Medical Pathology, Izmir Faculty of Medicine, University of Health Sciences, Izmir City Hospital, 35540 İzmir, Turkey; 13 Department of General Surgery, Izmir Faculty of Medicine, University of Health Sciences, Izmir City Hospital, 35540 İzmir, Turkey; 14 Department of Medical Pathology, Ege University, 35100 İzmir, Turkey; 15 Department of Medical Oncology, Acıbadem Hospital, 35330 İzmir, Turkey

**Keywords:** colorectal cancer, HER2, anti-EGFR therapy, RAS wild-type, predictive biomarker, prognostic factor

## Abstract

**Background:** Human epidermal growth factor receptor 2 (HER2) alterations have been implicated as mechanisms of resistance to anti-epidermal growth factor receptor (anti-EGFR) therapy in metastatic colorectal cancer (mCRC). We aimed to evaluate the predictive and prognostic significance of HER2 expression in patients with RAS wild-type mCRC in a real-world setting. **Methods:** We conducted a multicenter retrospective cohort study across ten oncology centers in Turkey, including patients with RAS wild-type mCRC treated between 2015 and 2022. Clinical outcomes, including progression-free survival (PFS) and overall survival (OS), were compared between HER2-positive and HER2-negative groups. Multivariable Cox proportional hazards models were used to identify independent predictors of survival outcomes. **Results:** Among 204 patients, 28 (13.7%) were HER2-positive. Baseline characteristics were generally comparable; however, HER2-positive patients showed a trend toward higher-grade tumors and were significantly less likely to receive anti-EGFR therapy. HER2-positive patients had significantly shorter PFS compared to HER2-negative patients (median 10 vs. 13 months; *p* = 0.006). In multivariable analysis, HER2 positivity remained an independent predictor of shorter PFS (HR 1.76, 95% CI 1.01–3.07; *p* = 0.045). In the subgroup of 144 patients receiving anti-EGFR therapy, HER2-positive patients also demonstrated significantly shorter PFS (median 9.0 vs. 14.0 months; *p* = 0.023). No significant differences in OS were observed between groups. **Conclusions:** HER2 positivity is associated with reduced response to anti-EGFR therapy and independently predicts shorter PFS in patients with RAS wild-type mCRC. These findings further support the role of HER2 as a clinically relevant biomarker in RAS wild-type mCRC, particularly in predicting response to anti-EGFR therapy, while highlighting the need for optimized patient selection strategies in the era of HER2-targeted treatments.

## 1. Introduction

Colorectal carcinoma (CRC) remains a significant global health burden, ranking as the second leading cause of cancer-related mortality worldwide [1]. Over the past two decades, the treatment landscape for metastatic colorectal cancer (mCRC) has substantially improved, particularly with the introduction of targeted therapies, such as anti-epidermal growth factor receptor (EGFR) monoclonal antibodies and anti-vascular endothelial growth factor (VEGF) agents [2,3,4,5,6]. The identification of RAS mutations has reshaped clinical practice by serving as a cornerstone biomarker for predicting resistance to anti-EGFR therapy [7,8,9].

However, primary and acquired resistance to anti-EGFR therapy remains a significant clinical challenge, with only 40–60% of RAS wild-type patients achieving objective responses [3,6]. Extensive research has revealed multiple molecular mechanisms underlying this resistance, including mutations in BRAF, PIK3CA, PTEN loss, and alterations in downstream signaling pathways [10,11,12,13,14,15,16]. Among these mechanisms, human epidermal growth factor receptor 2 (HER2) amplification and overexpression have emerged as clinically actionable biomarkers [17,18].

**Figure 1:**
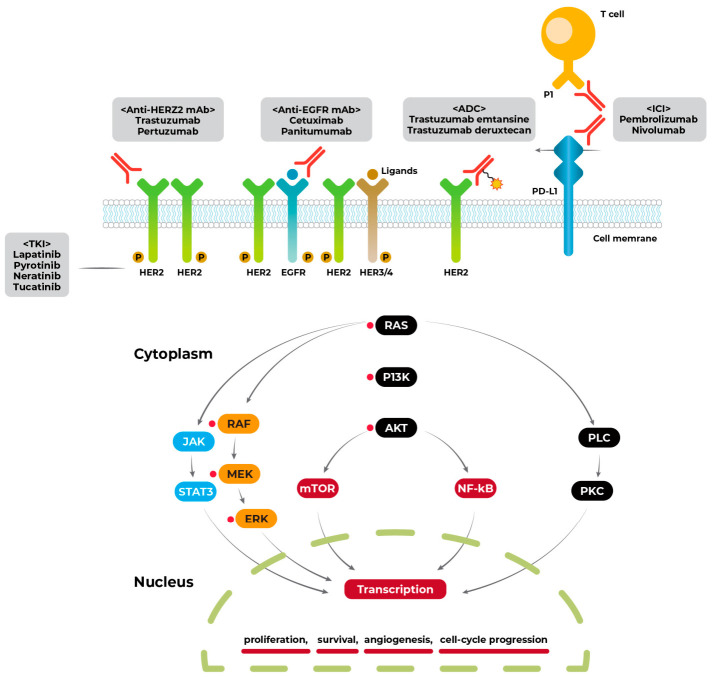
Signal transduction of the RAS/RAF/MEK/ERK pathway.

HER2 is a member of the ERBB family of receptor tyrosine kinases, which includes EGFR (HER1/ERBB1), HER3 (ERBB3), and HER4 (ERBB4) [19]. Unlike other members of this family, HER2 has no known ligand but functions as the preferred heterodimerization partner for other ERBB receptors (Figure 1) [20,21,22]. The formation of HER2/HER3 heterodimers activates downstream signaling cascades, including the PI3K/AKT and MAPK pathways, promoting cell proliferation, survival, and resistance to apoptosis [23,24]. In the context of anti-EGFR therapy, HER2 amplification or overexpression provides an alternative signaling pathway that bypasses EGFR blockade, thereby conferring resistance [25,26,27,28].

HER2 overexpression or amplification occurs in approximately 2–5% of mCRC cases, with higher prevalence (5–15%) reported in RAS wild-type populations. Importantly, the frequency of HER2 amplification appears to increase following exposure to anti-EGFR therapy, indicating mechanisms of acquired resistance [29,30,31]. Several retrospective studies and meta-analyses have demonstrated that HER2-positive status is associated with reduced response to anti-EGFR therapy and poor survival outcomes in RAS wild-type mCRC patients [32,33,34,35,36]. Beyond its role as a resistance biomarker, recent clinical trials evaluated HER2 as an actionable therapeutic target, reporting promising activity in mCRC patients. The HERACLES trial first demonstrated the efficacy of dual HER2 blockade with trastuzumab and lapatinib in HER2-positive, chemotherapy-refractory mCRC, achieving an ORR of 30% [37]. Subsequent trials, including MyPathway, MOUNTAINEER, and DESTINY-CRC01, have further established HER2-targeted therapy as an effective treatment option for HER2-positive mCRC [38,39,40,41].

Despite a growing body of literature, low HER2-positive incidence in CRC, considerable heterogeneity in testing methodologies across studies, and limited data from the era of effective HER2-targeted salvage therapies limit consistent interpretation of its utility in guiding treatment decisions. While recent international guidelines have increasingly recognized the clinical relevance of HER2 testing in mCRC, optimal testing algorithms for HER2 assessment are still under evaluation [42,43,44]. As a result, the predictive and prognostic significance of HER2 expression in RAS wild-type mCRC remains incompletely characterized, particularly in real-world clinical settings.

Therefore, we conducted a multicenter, retrospective cohort study across ten oncology centers in Turkey. Our objective was to assess the frequency and evaluate the predictive and prognostic relevance of HER2 expression in patients with RAS wild-type mCRC receiving treatment in routine clinical practice.

## 2. Materials and Methods

### 2.1. Study Design and Patient Population

This multicenter, retrospective cohort study was conducted across ten oncology centers in Turkey. We enrolled adult patients (≥18 years of age) with histologically confirmed RAS wild-type (absence of mutations in *KRAS* and NRAS exons 2, 3, and 4) CRC and received at least one line of systemic therapy for metastatic disease between January 2015 and December 2022. Patients with unknown HER2 status, incomplete medical records or loss to follow-up within 30 days of treatment initiation were excluded. The study protocol was approved by the Institutional Review Board of Bozyaka Training and Research Hospital (S122; 12 August 2020) and was conducted in accordance with the principles outlined in the Declaration of Helsinki. Given the retrospective design utilizing de-identified data, the requirement for individual informed consent was waived.

### 2.2. HER2 Testing and Classification

HER2 status was assessed by immunohistochemistry (IHC) and/or in situ hybridization (ISH) performed on formalin-fixed paraffin-embedded (FFPE) tumor tissue, obtained from either primary tumor resection specimens or metastatic site biopsies. HER2 assessment in this study was based on HERACLES diagnostic criteria, which define clinically actionable HER2 positivity using a combined IHC and ISH approach [37,45]. Accordingly, patients were categorized dichotomously as HER2-positive or HER2-negative. HER2 positivity was defined by either of the following conditions:

(1) IHC 3+ staining in more than 50% of tumor cells;
(2) IHC 3+ staining in 10–50% of tumor cells, or IHC 2+ staining in more than 50% of tumor cells, with concurrent FISH positivity defined as an ERBB2/CEP17 ratio ≥ 2.0 in ≥50% of cells.

IHC assessments were performed locally at each participating center by experienced pathologists using commercially available validated anti-HER2 antibodies (clone 4B5, Ventana Medical Systems, Tucson, AZ, USA; or clone A0485, Dako/Agilent, Glostrup, Denmark) on automated staining platforms (e.g., Ventana BenchMark Systems, Ventana Medical Systems, Tucson, AZ, USA; or Dako Autostainer platforms, Dako/Agilent Technologies, Glostrup, Denmark) in accordance with manufacturer protocols. Only membranous HER2 staining was considered for scoring, while cytoplasmic staining was regarded as non-specific and excluded.

Detailed stratification according to individual IHC categories (0, 1+, 2+, 3+) was not systematically available across all centers due to the retrospective and multicenter nature of the study. Moreover, given the lack of standardized interpretation and limited clinical relevance of intermediate IHC categories in mCRC, IHC stratification was not included in the analysis.

ISH testing was performed using dual-color fluorescence in situ hybridization (FISH) assays with probes targeting the ERBB2 gene and chromosome 17 centromere (CEP17) (e.g., PathVysion HER2 DNA Probe Kit, Abbott Molecular, Abbott Park, IL, USA, or equivalent validated assays), according to manufacturer instructions. A minimum of 50 non-overlapping tumor nuclei were evaluated per case to determine the ERBB2/CEP17 ratio.

### 2.3. Data Collection and Outcome Measures

Clinical and pathological data were retrospectively collected from electronic medical records at each participating center. Standardized case report form included patient and tumor characteristics (age, sex, performance status, primary tumor location, histological type, grade, number of metastatic sites, available molecular status), treatment history (systemic therapy regimens, anti-EGFR therapy use, primary tumor resection) and clinical outcomes.

Tumor grade was classified according to WHO criteria as Grade 1–2 (well to moderately differentiated) or Grade 3 (poorly differentiated). The number of metastatic sites was recorded at the time of initial metastatic diagnosis and categorized as single versus two or more sites. Performance status was assessed using the ECOG scale and patients were categorized as ECOG 0 or ECOG 1–2.

Progression-free survival (PFS) was defined as the interval from initiation of first-line systemic therapy to disease progression (according to RECIST v1.1 criteria) or death from any cause. Overall survival (OS) time was calculated from the diagnosis of metastatic disease to death from any cause. Patients without documented events were censored at the date of last follow-up.

### 2.4. Statistical Analysis

Descriptive statistics were used to summarize patient demographics and clinical characteristics. Continuous variables were presented as median with interquartile range (IQR). Comparisons between groups were performed using the Mann–Whitney U test for continuous variables and chi-square test or Fisher’s exact test for categorical variables. Spearman’s rank correlation analyses were performed to assess the association between HER2 status and histopathological features.

Survival curves were generated using the Kaplan–Meier method and compared using the log-rank test. The reverse Kaplan–Meier method was applied to estimate median follow-up time. Cox proportional hazards regression models were performed to evaluate associations between clinical and pathological variables and survival outcomes. Hazard ratios (HR) with 95% confidence intervals (CI) were calculated. Multivariable models were constructed using the enter method to minimize selection bias, including all clinically relevant variables with *p* < 0.05 in univariable analyses. Additional sensitivity analyses were performed using backward stepwise selection to assess the robustness of the analysis. BRAF mutational status was excluded from multivariable models given the limited number of BRAF-mutant cases, as well as the presence of missing data, which would have considerably diminished the sample size and compromised the statistical power of the multivariable analyses. Subgroup analyses were performed to evaluate the predictive value of HER2 status among patients who received anti-EGFR therapy.

All statistical tests were two-sided, and statistical significance was interpreted based on a *p*-value threshold of 0.05. Statistical analyses were performed using JAMOVI version 2.7.4 (Sydney, Australia) and SPSS version 30.0 (IBM Corp., Armonk, NY, USA).

## 3. Results

A total of 204 patients with RAS wild-type mCRC were included in the study. The median age at diagnosis of metastatic disease was 61 years (IQR: 51–68 years), and 139 patients (68.1%) were male. The majority of patients (62.7%) presented with ECOG PS of 1 or 2, while 37.3% had ECOG PS 0. Histologically, adenocarcinoma was the predominant subtype, accounting for 189 cases (92.6%). Of 190 patients with available data, 144 (75.8%) were classified as grade 1–2, and 46 (24.2%) as grade 3. With respect to disease presentation, 123 patients (60.3%) presented with de novo metastatic disease, while 81 patients (39.7%) had stage II–III disease. Anti-EGFR therapy was administered to 144 patients (70.6%).

Based on the predefined criteria, 28 patients (13.7%) were classified as HER2-positive and 176 (86.3%) as HER2-negative. Baseline demographic and clinicopathologic characteristics stratified by HER2 status are presented in Table 1. Age, sex, ECOG performance status, histological type, stage at diagnosis, BRAF mutation status, primary tumor location, and number of metastatic sites were comparable between the two cohorts (*p* > 0.05). Tumor grade demonstrated a trend toward higher-grade disease in the HER2-positive cohort, with 38.5% classified as grade 3 compared to 22.0% in the HER2-negative group (*p* = 0.068). To further explore the relationship between HER2 status and histopathological features associated with tumor aggressiveness, correlation analyses were performed. No significant correlations were observed between HER2 status and tumor grade (*p* = 0.117), lymphovascular invasion (*p* = 0.372), perineural invasion (*p* = 0.296), or histological subtype (*p* = 0.412). Notably, HER2-positive patients were significantly less likely to receive anti-EGFR therapy compared to their HER2-negative counterparts (53.6% vs. 73.3%, *p* = 0.033).

### 3.1. Survival Outcomes in the Overall Cohort

#### 3.1.1. Progression-Free Survival

**Figure 2:**
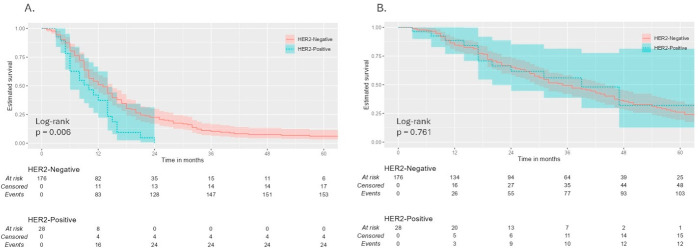
Kaplan–Meier survival curves in the overall cohort. (**A**) Progression-free survival by HER2 status. (**B**) Overall survival by HER2 status (n = 204).

The median follow-up duration for the entire cohort was 59 months (95% CI: 47.4–70.6 months). Follow-up duration differed between the two cohorts, with HER2-negative patients having a longer median follow-up of 65 months (95% CI: 50.5–79.5 months), whereas 36 months (95% CI: 27.5–44.5 months) for THE HER2-positive group. Over this follow-up period, 179 progression or death events were recorded, yielding an event rate of 87.7%. Median PFS (mPFS) for the entire cohort was 12.0 months (95% CI: 10.0–13.9). Landmark PFS rates were 49.2% (95% CI: 42.6–56.8%) at 12 months and 9.0% (95% CI: 5.6–14.3%) at 36 months. Kaplan–Meier analysis stratified by HER2 status revealed a statistically significant difference in PFS between the two groups, with HER2-positive patients demonstrating a notably shorter mPFS (10.0 vs. 13.0 months; log-rank *p* = 0.006) (Figure 2).

Univariable Cox regression analysis demonstrated that HER2 positivity was significantly associated with reduced PFS (HR: 1.84; 95% CI: 1.19–2.87; *p* = 0.006). The results of univariable and multivariable Cox regression analyses for PFS are summarized in Table 2. Beyond HER2 status, higher ECOG performance status (HR: 1.96; 95% CI: 1.43–2.69; *p* < 0.001), poorly differentiated histology (HR: 1.56; 95% CI: 1.09–2.23; *p* = 0.014), the presence of lymphovascular invasion (HR: 1.51; 95% CI: 1.05–2.17; *p* = 0.027), perineural invasion (HR: 1.41; 95% CI: 1.01–1.97; *p* = 0.044), and involvement of more than one metastatic site (HR: 1.35; 95% CI: 1.00–1.83; *p* = 0.049) emerged as significant adverse prognostic factors for PFS.

In multivariable analysis, HER2 positivity (HR: 1.76; 95% CI: 1.01–3.07; *p* = 0.045) and a higher ECOG performance status (1–2 vs. 0) (HR: 1.89; 95% CI: 1.31–2.74; *p* < 0.001) were identified as independent adverse prognostic factors for PFS. Sensitivity analyses using backward selection yielded consistent results, with HER2 status remaining an independent prognostic factor for PFS (Table S1).

#### 3.1.2. Overall Survival

The median OS (mOS) for the entire cohort was 37.0 months (95% CI: 29.8–44.1 months), with landmark survival rates of 85.0% (95% CI: 80.0–90.2%), 50.1% (95% CI: 43.0–58.3%), and 26.5% (95% CI: 19.9–35.3%) at 12, 36, and 60 months, respectively. Kaplan–Meier analysis showed no significant difference in OS between HER2-positive and HER2-negative patients (log-rank *p* = 0.761), with a mOS of 39 months (95% CI: 23.6–54.5 months) versus 35 months (95% CI: 27.3–42.7 months) (Figure 2).

For OS, univariable analysis identified several significant adverse prognostic factors, including older age (HR: 1.57; 95% CI: 1.09–2.24; *p* = 0.014), higher ECOG performance status scores (HR: 3.21; 95% CI: 2.16–4.78; *p* < 0.001), poorly differentiated histology (HR: 1.53; 95% CI: 1.01–2.32; *p* = 0.045), lymphovascular invasion (HR: 1.66; 95% CI: 1.07–2.58; *p* = 0.025), perineural invasion (HR: 1.69; 95% CI: 1.14–2.54; *p* = 0.010), and involvement of more than one metastatic site (HR: 2.18; 95% CI: 1.51–3.14; *p* < 0.001) (Table 3). HER2 status was not significantly associated with OS (HR: 0.91; 95% CI: 0.50–1.66; *p* = 0.763). Of note, de novo metastatic disease was paradoxically associated with a more favorable OS compared with metastatic progression from previously early-stage disease (HR: 0.66; 95% CI: 0.47–0.94; *p* = 0.021).

In the multivariable analysis, ECOG performance status of 1 or 2 (HR: 3.29; 95% CI: 2.01–5.39; *p* < 0.001) and involvement of more than one metastatic site (HR: 1.74; 95% CI: 1.12–2.68; *p* = 0.013) were identified as independent adverse prognostic factors for OS. In the multivariable analysis, ECOG performance status of 1 or 2 (HR: 3.29; 95% CI: 2.01–5.39; *p* < 0.001) and involvement of more than one metastatic site (HR: 1.74; 95% CI: 1.12–2.68; *p* = 0.013) were identified as independent adverse prognostic factors for OS. Sensitivity analyses using backward stepwise selection produced consistent results, confirming ECOG performance status and metastatic burden as independent prognostic factors and additionally identifying perineural invasion, whereas HER2 status was not retained in the final model (Table S2).

### 3.2. Survival Outcomes in Patients Receiving Anti-EGFR Therapy

**Figure 3:**
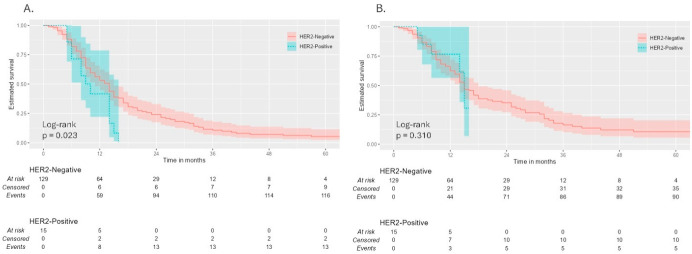
Kaplan–Meier survival curves in patients receiving anti-EGFR therapy. (**A**) Progression-free survival by HER2 status. (**B**) Overall survival by HER2 status (n = 144).

Among the 144 patients (70.6%) who received anti-EGFR therapy, 15 (10.4%) were HER2-positive and 129 (89.6%) were HER2-negative. In this subgroup, HER2-positive patients demonstrated significantly shorter PFS compared with HER2-negative patients (median 9.0 vs. 14.0 months; *p* = 0.023) (Figure 3). OS did not differ significantly between the two groups (median 47.0 vs. 39.0 months; *p* = 0.310) (Figure 3).

## 4. Discussion

In this multicenter retrospective cohort study involving 204 patients with RAS wild-type mCRC, we aimed to evaluate the predictive and prognostic significance of HER2 expression in a real-world clinical setting. Our findings reveal several critical insights regarding HER2 status and its implications for patient outcomes.

The prevalence of HER2 positivity observed in our study (13.7%) substantially exceeds that documented in molecularly unselected mCRC populations (2–4%) [30,46]. Given that our study population was restricted to RAS wild-type tumors, this enrichment of HER2 positivity suggests potential biological crosstalk between the RAS and HER2 signaling pathways, supporting the hypothesis that HER2 amplification may function as an alternative oncogenic driver in the absence of RAS mutations [17,25,47,48]. Consistent with the principle of pathway redundancy within the ERBB signaling network, tumors lacking canonical RAS activation may compensate by exploiting upstream receptor amplification to sustain proliferative signaling [10,47]. Beyond this molecular perspective, selection bias may further account for this enrichment, as HER2 testing may have been performed preferentially in a clinically pre-selected population with suspected HER2 positivity. Moreover, the wide variability in HER2 positivity rates reported across the literature is largely attributable to heterogeneity in testing methodologies [49,50,51]. The optimal diagnostic criteria for HER2 positivity in mCRC remain incompletely standardized, with ongoing international efforts aimed at harmonizing testing methodologies and interpretation guidelines [42]. Such standardization will be critical for consistent patient identification and for enabling reliable cross-study comparisons. Within this context, the HER2 positivity rate observed in our cohort falls within the 5–15% range previously reported in RAS wild-type mCRC populations and is consistent with the existing literature [46,48,52].

In our study, HER2-negative patients demonstrated significantly longer PFS than HER2-positive patients. Multivariable Cox regression analyses confirmed that HER2 positivity remained an independent adverse prognostic factor for PFS. Furthermore, among patients who received anti-EGFR therapy, HER2-positive individuals demonstrated significantly shorter PFS compared to their HER2-negative counterparts. These findings are consistent with prior evidence and further support the hypothesis that HER2 amplification confers resistance to EGFR-directed therapy in RAS wild-type mCRC [17,25,35,48]. Taken together, these results suggest that HER2 status warrants prospective evaluation as a stratification variable in future clinical trials and may inform the selection of patients most likely to benefit from HER2-targeted therapeutic strategies. A noteworthy finding of our study is that HER2-positive patients were significantly less likely to receive anti-EGFR therapy compared to their HER2-negative counterparts. This practice pattern likely reflects growing awareness of the negative predictive value of HER2 amplification for anti-EGFR therapy based on emerging clinical trial data and retrospective analyses and validates the clinical relevance of routine HER2 testing [25,48]. Despite this inherent selection bias, a significant PFS difference within the anti-EGFR therapy subgroup reinforces our findings regarding the predictive value of HER2 positivity for anti-EGFR therapy response.

In contrast to its significant impact on PFS, HER2 positivity was not significantly associated with OS in either the overall cohort or the anti-EGFR therapy subgroup. This pattern is characteristic of a predictive biomarker that modulates response to a specific therapeutic intervention without fundamentally altering long-term prognosis [53,54]. This dissociation between PFS and OS effects has been reported in previous studies and may reflect the availability of effective subsequent therapies [35,55,56]. Additionally, the limited number of HER2-positive patients in our cohort, combined with a shorter follow-up duration in this subgroup, may have constrained statistical power to detect a modest OS difference.

HER2 amplification has been implicated in more aggressive tumor biology, characterized by higher histological grade and enhanced metastatic potential that may adversely affect clinical outcomes [57,58]. In our cohort, a numerical trend toward higher-grade tumors was observed in HER2-positive patients; however, this difference did not reach statistical significance. Moreover, correlation analyses did not demonstrate a significant association between HER2 status and conventional histopathological features associated with tumor aggressiveness, including tumor grade, lymphovascular invasion, perineural invasion, and histological subtype. These findings suggest that the clinical impact of HER2 may not be mediated through traditional morphological characteristics, but rather through distinct molecular mechanisms. Consistent with this, accumulating evidence indicates that oncogenic driver alterations, including HER2, exert their effects predominantly through dysregulation of key signaling pathways such as RAS/MAPK and PI3K/AKT [59,60]. This interpretation is further supported by multi-omics integration studies, which highlight that oncogenic driver events function within complex, multi-layered biological systems, where interactions across genomic, transcriptomic, and epigenetic levels collectively determine tumor behavior rather than isolated molecular alterations.

Contrary to the existing literature, OS was worse in metachronous patients despite comparable PFS. Most reports calculate OS from the time of primary diagnosis, leading to elevated survival rates [61,62]. Although chemoresistance related to prior adjuvant therapy has been proposed as an explanation, robust supporting evidence remains lacking [63]. The methodological difference most likely accounts for this discordance, as OS in the present study was calculated from the date of metastatic diagnosis. Moreover, the comparable PFS further supports biological similarity at the metastatic stage [64,65].

For patients with HER2-positive, RAS wild-type mCRC, anti-EGFR therapy may not represent the optimal treatment choice. While anti-EGFR therapy may still provide some benefit, these patients should be prioritized for HER2-targeted therapies, which have demonstrated significant clinical activity in this molecular subgroup. Multiple clinical trials have established the efficacy of HER2-targeted therapies in HER2-positive mCRC, including dual HER2 blockade with trastuzumab plus lapatinib or pertuzumab, and antibody-drug conjugates such as trastuzumab deruxtecan [38,39,40]. In settings where HER2-targeted therapies are not accessible, chemotherapy with bevacizumab may represent a reasonable alternative to anti-EGFR therapy, as our data suggest that HER2 positivity specifically predicts resistance to EGFR inhibition rather than conferring a broadly adverse prognosis.

Despite the observational design of our study, the link between HER2 positivity and reduced responsiveness to anti-EGFR therapy is biologically compelling and strongly supported by prior mechanistic data. HER2 amplification has been shown to activate alternative signaling pathways within the ERBB network, enabling downstream signaling independent of EGFR and thereby contributing to resistance to EGFR-directed therapies [59]. The late divergence of PFS curves likely reflects the time-dependent emergence of HER2-driven resistance. While initial responses may be comparable due to suppression of dominant tumor clones, intratumoral heterogeneity facilitates the gradual expansion of HER2-amplified resistant subclones over time. In this context, HER2 amplification may function as both a primary and acquired resistance mechanism, as supported by genomic and ctDNA analyses demonstrating its presence in nonresponsive tumors and its emergence at disease progression [66]. Multi-omics analyses across tumor types further support the role of HER2 as a potential driver-level alteration within compensatory signaling pathways, particularly in the absence of canonical RAS activation [60,67]. In addition, intratumoral heterogeneity and variability in HER2 expression, as well as technical challenges in HER2 assessment, may contribute to differences in reported prevalence and treatment response across studies. While the retrospective nature of our study precludes direct molecular validation, these data provide a biological context for our findings and support the interpretation that HER2 status reflects a clinically relevant mechanism of treatment resistance rather than a purely prognostic association.

Current guidelines from the European Society for Medical Oncology (ESMO) and the National Comprehensive Cancer Network (NCCN) recommend HER2 testing in this population, particularly for patients with treatment-refractory disease [44,68]. Although HER2 testing is already incorporated into routine clinical practice in many tertiary and academic centers, our findings reinforce its clinical relevance, particularly in identifying patients less likely to benefit from anti-EGFR therapy. However, the lack of standardized testing protocols and the variability in HER2 positivity rates across studies necessitate further research to establish optimal testing algorithms and diagnostic criteria.

Several limitations of this study warrant acknowledgment and careful consideration when interpreting the results. As a retrospective observational study, our findings are subject to inherent limitations, including selection bias, unmeasured confounders, and incomplete data capture. The heterogeneity in treatment regimens with patients receiving various chemotherapy backbones reflects real-world clinical practice but may introduce confounding effects into survival analyses. Furthermore, detailed information regarding subsequent lines of therapy and the utilization of HER2-targeted agents was unavailable. In addition, the timing of HER2 testing was not uniform across the cohort, and HER2 status was not consistently available at the time of treatment initiation. As a result, treatment decisions were not systematically guided by HER2 status, supporting the interpretation that the observed associations may not solely reflect a treatment-driven predictive effect. Nevertheless, increasing awareness of HER2 status over the study period may have influenced treatment selection in a subset of patients, potentially contributing to the lower use of anti-EGFR therapy among HER2-positive cases. The absence of systematic evaluation of other potentially relevant biomarkers, such as BRAF mutations and microsatellite instability status, represents an additional limitation, as these alterations are known to influence tumor biology and prognosis. This limitation may introduce residual confounding and hinder the ability to fully contextualize the independent contribution of HER2 status within a broader molecular landscape. Although adherence to standardized HERACLES criteria for HER2 assessment could have minimized inter-institutional variability, the lack of centralized pathology review, together with the retrospective, multicenter design, precluded formal assessment of interobserver agreement and constrained the availability and comparability of detailed IHC stratification across centers.

Our study provides robust real-world evidence supporting the clinical relevance of HER2 in RAS wild-type mCRC, with several distinguishing strengths. Leveraging a large, multicenter cohort from ten oncology centers, it reflects contemporary treatment patterns and biomarker utilization in routine practice, enhancing the generalizability of our findings. By focusing on a molecularly homogeneous RAS wild-type population and incorporating clinically meaningful subgroup analyses, particularly among patients treated with anti-EGFR therapy, we demonstrate the impact of HER2 status on progression outcomes in real-world settings, reinforcing its role as a clinically relevant biomarker beyond controlled trial environments. Looking forward, our findings underscore the importance of integrating HER2 testing into routine diagnostic workflows and highlight the need for prospective, biomarker-driven studies to further clarify its predictive role and optimize patient selection for targeted strategies.

## 5. Conclusions

In conclusion, this multicenter study demonstrates that HER2 positivity is associated with reduced responsiveness to anti-EGFR therapy in patients with RAS wild-type mCRC, supporting its role as a clinically relevant biomarker in routine practice. While our findings reinforce the importance of incorporating HER2 testing into clinical decision-making, they do not provide direct evidence to support the prioritization of HER2-targeted therapies, given the lack of data on subsequent treatment lines. Further prospective studies with larger cohorts and standardized methodologies are warranted to validate these observations and to better define the optimal integration of HER2-directed strategies into treatment algorithms.

## Figures and Tables

**Table 1:** Baseline Patient Characteristics by HER2 Status.

Variables	Total (n = 204)	HER2-Positive (n = 28)	HER2-Negative (n = 176)	*p*-Value
Age, years Median (IQR) <65 ≥65	61 (51–68) 133 (65.2) 71 (34.8)	58 (48–69.8) 18 (64.3) 10 (35.7)	61 (51.6–67) 116 (65.3) 61 (34.7)	0.997 ^†^ 0.913 ^a^
Sex Female Male	65 (31.9) 139 (68.1)	12 (42.9) 16 (57.1)	53 (30.1) 123 (69.9)	0.179 ^a^
ECOG PS 0 1–2	76 (37.3) 128 (62.7)	11 (39.3) 17 (60.7)	65 (36.9) 111 (63.1)	0.811 ^a^
Histological type Adenocarcinoma Other *	189 (92.6) 15 (7.4)	27 (96.4) 1 (3.6)	162 (92.0) 14 (8.0)	0.409 ^b^
Histological grade Grade 1–2 Grade 3	144 (75.8) 46 (24.2)	16 (61.5) 10 (38.5)	128 (78.0) 36 (22.0)	0.068 ^a^
Stage at diagnosis II–III IV	81 (39.7) 123 (60.3)	9 (32.1) 19 (67.9)	72 (40.9) 104 (59.1)	0.379 ^a^
Lymphovascular invasion Negative Positive	52 (29.7) 123 (70.3)	9 (37.5) 15 (62.5)	43 (28.5) 108 (71.5)	0.369 ^a^
Perineural invasion Negative Positive	72 (42.1) 99 (57.9)	12 (52.2) 11 (47.8)	60 (40.5) 88 (59.5)	0.293 ^a^
BRAF status Wild-type Mutant	138 (87.9) 19 (12.1)	27 (96.4) 1 (3.6)	111 (86.0) 18 (14.0)	0.200 **^b^**
Primary tumor resection No Yes	39 (19.5) 161 (80.5)	5 (17.9) 23 (82.1)	34 (19.8) 138 (80.2)	0.813 ^a^
Tumor location Rectum Right Colon Left Colon	51 (25.2) 51 (25.2) 100 (49.5)	8 (28.6) 5 (17.9) 15 (53.6)	43 (24.7) 46 (26.4) 85 (48.9)	0.621 ^a^
Anti-EGFR therapy No Yes	60 (29.4) 144 (70.6)	13 (46.4) 15 (53.6)	47 (26.7) 129 (73.3)	**0.033 ^a^**
Number of metastatic sites 1 site ≥2 sites	93 (47.0) 105 (53.0)	14 (51.9) 13 (48.1)	79 (46.2) 92 (53.8)	0.584 ^a^
Adjuvant treatment No Yes	135 (66.5) 68 (33.5)	21 (75.0) 7 (25.0)	114 (65.1) 61 (34.9)	0.305 ^a^

* Other included, mucinous carcinoma (n = 12) and signet ring cell carcinoma (n = 3). Data are presented as n (%) for categorical variables and median (IQR) for continuous variables. *p*-values calculated using ^a^ Chi-square or ^b^ Fisher’s exact test and ^†^ Mann–Whitney U tests. Bold values indicate statistically significant differences at a level of ≤0.05. ECOG PS, Eastern Cooperative Oncology Group Performance Status; EGFR, epidermal growth factor receptor; HER2, human epidermal growth factor receptor 2; IQR, Interquartile range.

**Table 2:** Univariable and Multivariable Cox Regression Analysis for Progression-Free Survival.

Variables	Univariable HR [95% CI]	*p*-Value	Multivariable HR [95% CI]	*p*-Value
**Age group** <65 ≥65	Ref 1.05 [0.77–1.43]	0.765	0.95 [0.67–1.35]	0.771
**Sex** Female Male	Ref 0.81 [0.59–1.11]	0.189	–	–
**HER2 status** Negative Positive	Ref 1.84 [1.19–2.87]	**0.006**	Ref 1.76 [1.01–3.07]	**0.045**
**ECOG PS** 0 1–2	Ref 1.96 [1.43–2.69]	**<0.001**	Ref 1.89 [1.31–2.74]	**<0.001**
**Histological type** Adenocarcinoma Other	Ref 1.21 [0.66–1.90]	0.672	–	–
**Histological grade** Grade 1–2 Grade 3	Ref 1.56 [1.09–2.23]	**0.014**	Ref 1.45 [0.96–2.18]	0.078
**Stage at diagnosis** II–III IV	Ref 1.05 [0.78–1.42]	0.735	–	–
**Lymphovascular invasion** Negative Positive	Ref 1.51 [1.05–2.17]	**0.027**	Ref 1.40 [0.91–2.15]	0.118
**Perineural invasion** Negative Positive	Ref 1.41 [1.01–1.97]	**0.044**	Ref 1.19 [0.81–1.76]	0.365
**Primary tumor resection** No Yes	Ref 0.71 [0.48–1.04]	0.077	–	–
**Tumor Location** Rectum Right Colon Left Colon	Ref 1.19 [0.79–1.79] 1.21 [0.84–1.73]	0.403 0.310	–	–
**Anti-EGFR therapy** No Yes	Ref 0.89 [0.64–1.23]	0.474	–	–
**Number of metastatic sites** 1 site ≥2 sites	Ref 1.35 [1.00–1.83]	**0.049**	Ref 1.21 [0.85–1.71]	0.300
**Adjuvant therapy** No Yes	Ref 0.88 [0.65–1.21]	0.431		

Other included, mucinous carcinoma (n = 12) and signet ring cell carcinoma (n = 3). Data are presented as hazard ratios (HR) and 95% confidence intervals (95%CI). Bold values indicate statistically significant differences at a statistical significance level of ≤0.05. ECOG PS, Eastern Cooperative Oncology Group performance status; EGFR, epidermal growth factor receptor; HER2, human epidermal growth factor receptor 2.

**Table 3:** Univariable and Multivariable Cox Regression Analysis for Overall Survival.

Variables	Univariable HR [95% CI]	*p*-Value	Multivariable HR [95% CI]	*p*-Value
**Age, years** <65 ≥65	Ref 1.57 [1.09–2.24]	**0.014**	Ref 1.38 [0.92–2.09]	0.124
**Sex** Female Male	Ref 1.41 [0.95–2.10]	0.094	–	–
**HER2 status** Negative Positive	Ref 0.91 [0.50–1.66]	0.763	–	–
**ECOG PS** 0 1–2	Ref 3.21 [2.16–4.78]	**<0.001**	Ref 3.29 [2.01–5.39]	**<0.001**
**Histological type** Adenocarcinoma Other *	Ref 1.07 [0.57–2.01]	0.843	–	–
**Histological grade** Grade 1–2 Grade 3	Ref 1.53 [1.01–2.32]	**0.045**	Ref 1.45 [0.88–2.37]	0.142
**Stage at diagnosis** II–III IV	Ref 0.66 [0.47–0.94]	**0.021**	Ref 0.77 [0.51–1.19]	0.239
**Lymphovascular invasion** Negative Positive	Ref 1.66 [1.07–2.58]	**0.025**	Ref 1.40 [0.88–2.60]	0.136
**Perineural invasion** Negative Positive	Ref 1.69 [1.14–2.54]	**0.010**	Ref 1.48 [0.93–2.35]	0.099
**Primary tumor resection** No Yes	Ref 0.75 [0.45–1.26]	0.274	–	–
**Tumor location** Rectum Right Colon Left Colon	Ref 1.31 [0.83–2.07] 0.84 [0.55–1.29]	0.244 0.431	–	–
**Anti-EGFR therapy** No Yes	Ref 0.722 [0.49–1.08]	0.110	–	–
**Number of metastatic sites** 1 site ≥2 sites	Ref 2.18 [1.51–3.14]	**<0.001**	Ref 1.74 [1.12–2.68]	**0.013**
**Adjuvant therapy** No Yes	Ref 1.24 [0.86–1.77]	0.248	–	–

* Other included, mucinous carcinoma (n = 12) and signet ring cell carcinoma (n = 3). Data are presented as hazard ratios (HR) and 95% confidence intervals (95%CI). Bold values indicate statistically significant differences at a statistical significance level of ≤0.05. ECOG PS, Eastern Cooperative Oncology Group performance status; EGFR, epidermal growth factor receptor; HER2, human epidermal growth factor receptor 2.

## Data Availability

The datasets generated and analyzed during the current study are available from the corresponding author upon reasonable request.

## Supplementary Materials

The following supporting information can be downloaded at https://www.mdpi.com/article/10.3390/jcm15103979/s1, Table S1. Multivariable Cox regression analysis using backward stepwise selection for progression-free survival (PFS). Table S2. Multivariable Cox regression analysis using backward stepwise selection for overall survival (OS).
