# Supplementary material for: Prognostic and Treatment-Specific Predictive Implications of HER2 Expression in RAS Wild-Type Metastatic Colorectal Cancer: A Multicenter Retrospective Real-World Study
Source: J Clin Med. 2026 May 21;15(10):3979. doi: 10.3390/jcm15103979 (PMC13207513; doi:10.3390/jcm15103979)
Supplement: Supplementary file 1 [file jcm-15-03979-s001.zip › jcm-4299248-supplementary.pdf]

Table S1. Multivariable Cox regression analysis using backward stepwise selection for progression-free survival (PFS)

Variable	HR [95% CI]	p-value
HER2 status (Negative vs. Positive)	2.30 [1.32–4.00]	0.003
ECOG PS (0 vs. 1-2)	2.09 [1.43–3.05]	<0.001
Perineural invasion (Negative vs. Positive)	1.47 [1.02–2.10]	0.037
Anti-EGFR therapy (No vs. Yes)	1.45 [0.94–2.23]	0.092

Variables were selected using backward stepwise selection based on the likelihood ratio test. Data are presented as hazard ratios (HR) and 95% confidence intervals (95%CI).

ECOG PS; Eastern Cooperative Oncology Group performance status, EGFR; epidermal growth factor receptor; HER2; human epidermal growth factor receptor 2

Table S2. Multivariable Cox regression analysis using backward stepwise selection for overall survival (OS)

Variable	HR [95% CI]	p-value
ECOG PS (0 vs. 1-2)	3.34 [2.01–5.56]	<0.001
Perineural invasion (Negative vs. Positive)	1.88 [1.20–2.93]	0.006
Number of metastatic sites (1 vs. ≥2 sites)	1.82 [1.18–2.81]	0.006
Stage at diagnosis (II-III vs. IV)	0.28 [0.09–0.84]	0.023
Adjuvant treatment history (No vs. Yes)	0.32 [0.11–0.93]	0.037

Variables were selected using backward stepwise selection based on the likelihood ratio test. Data are presented as hazard ratios (HR) and 95% confidence intervals (95%CI).

ECOG PS; Eastern Cooperative Oncology Group performance status
